# Epidemiology and Clinical Outcomes of HIV Infection in South-Central China: A Retrospective Study From 2003 to 2018

**DOI:** 10.3389/fpubh.2022.902537

**Published:** 2022-06-09

**Authors:** Tingting Yue, Pan Zhang, Yuantao Hao, Jianmei He, Jun Zheng, Erik De Clercq, Guangdi Li, Yaxiong Huang, Fang Zheng

**Affiliations:** ^1^Hunan Provincial Key Laboratory of Clinical Epidemiology, Xiangya School of Public Health, Central South University, Changsha, China; ^2^Department of Medical Statistics and Epidemiology, School of Public Health, Sun Yat-sen University, Guangzhou, China; ^3^Hunan Center for Disease Control and Prevention, Changsha, China; ^4^Department of Microbiology, Immunology and Transplantation, Rega Institute for Medical Research, KU Leuven, Leuven, Belgium; ^5^Hunan Children's Hospital, Changsha, China; ^6^Department of Infectious Disease, The First Hospital of Changsha, Changsha, China

**Keywords:** HIV, epidemiology, elderly males, heterosexual transmission, HIV-associated fatality

## Abstract

**Objective:**

HIV epidemiology in South-Central China is rarely reported. This study aims to characterize epidemiological and clinical features of HIV-infected patients in Hunan Province, located in South-Central China, for better management of HIV infections.

**Methods:**

This retrospective study retrieved multi-center records of laboratory-confirmed HIV-infected patients in Hunan province. Information on HIV-associated mortality and antiretroviral therapies was also collected.

**Results:**

Among 34,297 patients diagnosed with HIV infections from 2003 to 2018, 73.9% were males, 41.3% were older adults (≥50 years), and 71.2% were infected by heterosexual transmission. Despite a slow growth of new HIV infections in the overall population, annual percentages of HIV infections increased in older males (85.3% through heterosexual transmission) and young patients <30 years (39.9% through homosexual transmission). At baseline, serum levels of CD4+ T-cell counts were lower in older adults (191.0 cells/μl) than in young patients (294.6 cells/μl, *p*-value < 0.0001). A large proportion (47.2%, *N* = 16,165) of HIV-infected patients had advanced HIV disease (CD4+ T-cell counts < 200 cells/μl) from 2003 to 2018. All-cause mortality (57.0% due to AIDS-related illnesses) was reported among 4411 HIV-infected patients, including 2619 older adults. The 10-year survival rate was significantly lower in elderly males than in other patients (59.0 vs. 78.4%, *p*-value < 0.05).

**Conclusions:**

Elderly males are prone to HIV infections with a high risk of HIV-associated fatality. Our findings support early prevention and critical care for elderly populations to control HIV infections.

## Introduction

Human immunodeficiency virus (HIV) infection causes progressive damage to the immune system, characterized by massive depletion of CD4+ T-cells, sustained immune activation, and systemic inflammation (1). As of today, more than 37 million patients are living with HIV globally and ~1.1 million cases are living in China (http://www.unaids.org). Since the introduction of antiretroviral therapies (ART) in the 1980s (2, 3), the mortality among people living with HIV has decreased significantly (4) and the life expectancy of HIV-infected patients has improved dramatically (5, 6). Most antiretroviral regimens consist of three oral compounds, including two nucleoside reverse transcriptase inhibitors (NRTIs) plus either one non-nucleoside reverse transcriptase inhibitor (NNRTI), one integrase inhibitor, or one protease inhibitor boosted by ritonavir or cobicistat (7–9). Although many highly active antiretroviral therapies have been approved in the past decades (3, 9), an effective drug or vaccine remains unavailable to “cure” HIV infections (10). To curb HIV infections, it remains important for epidemiological studies to identify high-risk populations in different countries and regions.

Since the first report of HIV infection in China in 1989 (11), HIV has spread across the country in the past decades. In 2018, 64,170 patients and 18,780 HIV-related deaths were reported in China (12). The overall incidence of HIV in China was not high but on the rise over time, and the fatality rate of HIV was ranked among the top five notifiable infectious diseases (13). A series of prevention and treatment strategies have been implemented in China (14). In 2003, China launched the “four free and one care” policy for HIV (free treatment, free voluntary counseling and testing, free prevention of mother-to-child transmission, free schooling for AIDS orphans, and social care for HIV-infected patients) (15). In 2011, the “five expand six strengthen” policy was implemented in China to strengthen the national coverage, including HIV testing and surveillance, health education, blood administration and safety, prevention of mother-to-child transmission, antiretroviral therapies, as well as health care and social support (16). Since 2003, the National Free Antiretroviral Treatment Program (NFATP) has been implemented in China, despite the limited variety of free antiretroviral drugs. With the support of NFATP, the ART has undergone continuous evaluation and improvement (17). Over the past decades, China has made substantial achievements in the prevention and treatment of HIV. However, it remains a challenge to prevent and control HIV infections in China because of dynamic transportation, economic development, and social and cultural dynamics (16, 18). In the past 20 years, the incidences of intravenous drug use, mother-to-child transmission, and blood transmission decreased substantially in China (19), but an increase in HIV infections can be found in the elderly population (14, 20) and young students (21). Sexual transmission remains the primary route of HIV transmission in China (22), but the incidences of homosexual transmission are growing in recent years (23–25). Furthermore, the geographical distribution of HIV infections varies in different provinces and regions in China (14). For instance, homosexual transmission in a province in northeast China accounted for the majority (69%) of HIV infections from 2011 to 2012 (26). Another epidemiological feature is that 77.1% of HIV infections in Southeastern China were found in patients aged 19–50 years (27). Taken together, it remains important to explore local epidemiological features to control HIV infections.

Hunan province, located in South-Central China, is known for its large population and is one of the most popular tourist destinations in China. Since the first report of HIV infection in Hunan Province in 1992, there is a growing trend of HIV incidences. However, no report has published epidemiological and clinical features of HIV-infected patients in Hunan province. Here, we analyzed a large-scale cohort of 32,419 HIV-infected patients who were diagnosed from 2003 to 2018 in Hunan province. Our study will provide the first comprehensive survey of the temporal trend, spatial distribution, and population characteristicsof HIV infection, virological responses, and survival status of HIV-infected patients who received standard ART. This study will shed light on the temporal trend of HIV infections in a high-risk population for better management and prevention of HIV infections in Hunan province.

## Methods

### Study Design and Patients

We performed a retrospective study to collect epidemiological and clinical records of HIV-infected patients based on the official database from the Centers for Disease Control in Hunan province. This database includes patient information on demographic records, serum blood biomarkers, ART records, HIV-associated death dates, and virological responses. All patients were confirmed to have laboratory-confirmed HIV infection during the period from 2003 to 2018. According to the national regulation, any HIV-positive case should be officially reported to the Centers for Disease Control in China. Serum levels of CD4+ T-cells and CD8+ T cells were assessed using the BD FACSCalibur flow cytometer. HIV viral loads were quantified using the COBAS^®^ AmpliPrep instrument. This retrospective study was conducted under the Helsinki Declaration and was approved by the Ethics Committees of The First Hospital of Changsha (Approval ID: 202160).

### Definitions

Failure of immune reconstitution was defined by the persistent CD4 level <100 cells/μl (28). As described previously (29), HIV-infected patients experienced the status of poor immune reconstruction if two conditions were fulfilled: (i) CD4+ T-cell counts < 350 cells/μl; and (ii) CD4+ T-cell increases < 100 cells/μl in those patients with HIV RNA <50 copies/ml for more than 1 year. Advanced HIV disease was defined as presenting for HIV care with CD4+ T-cell counts <200 cells/μL or WHO stage 3/4 conditions.

### Statistical Analysis

Categorical variables were presented as frequencies and percentages, while continuous variables were analyzed by mean and standard deviations. To analyze the temporal trend of new HIV infections per year, we used Joinpoint regression models (https://surveillance.cancer.gov/joinpoint/) to calculate the annual percentage changes (APC) as well as the trend test of APC. The grid search method was used to determine the joinpoint, and permutation tests were used to select the optimal model. Paired-*t*-tests were used to detect differences in CD4 levels before and after treatment. Chi-square tests were applied to compare the proportion of virological responses in patients receiving different ART. Welch's ANOVA methods were used to compare the means of multiple groups, while Games-Howell tests were used for pairwise comparisons between groups. *P*-values of multiple comparisons were corrected by the Holm method. The survival rate was estimated by the Kaplan-Meier method, while log-rank tests were used to evaluate any difference. Our statistical analyses used the pairwise deletion approach to handle missing data. All statistical analyses were descriptive, and no random sampling was conducted. A statistical significance was considered when a *p*-value was below 0.05. We performed statistical analyses using the Joinpoint Regression Program 4.9.0.0, R x64 4.1.0, and GraphPad prism 8.0.1.

## Results

**Figure 1:**
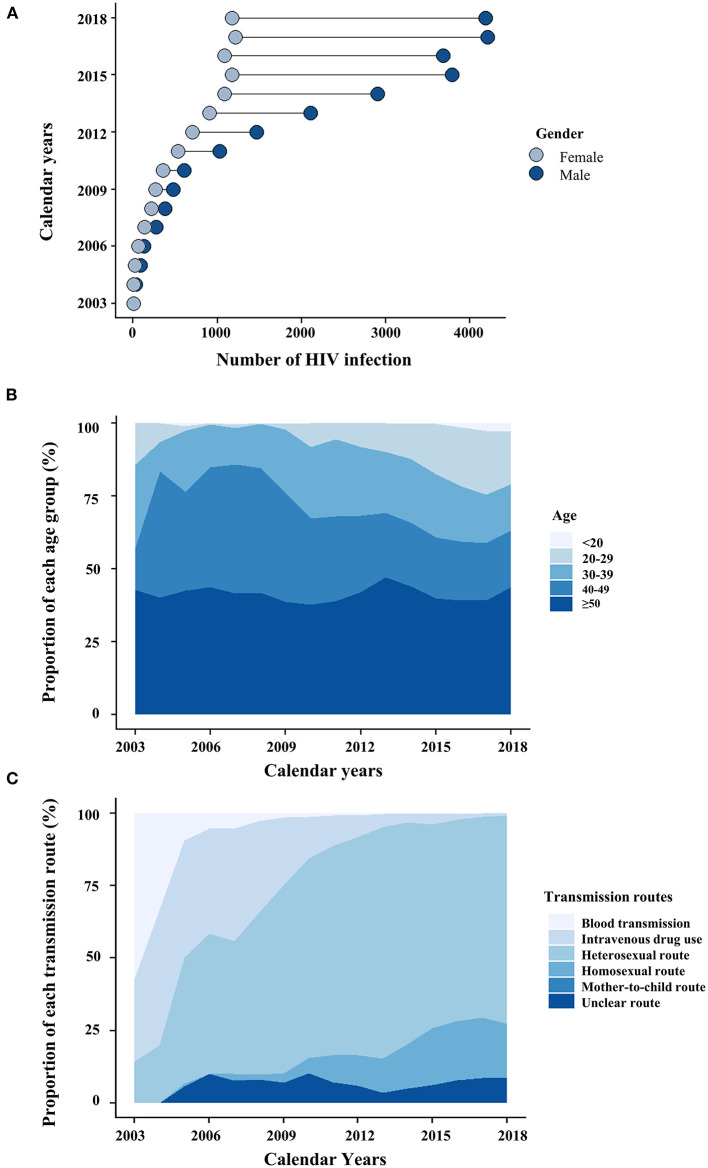
The dynamics of HIV-infected patients are categorized by gender **(A)**, age **(B)**, and transmission routes **(C)**.

From 2003 to 2018, a total of 34,297 HIV-infected patients were treated at the designated hospitals in Hunan province. Patient information is summarized in Table 1. Gender disparity was observed with a large proportion of male patients (*N* = 25,338, 73.9%). The number of HIV infections in both males and females increased over time, whereas the increasing numbers grew faster in males (Figure 1A). At the time of their first HIV-positive diagnosis, the youngest and oldest patients were 7 and 95 years of age, respectively. The median age at the first diagnosis of HIV infection was 46 years old. Among all age groups, patients aged ≥50 years accounted for the largest proportion at all times, the number of HIV-infected patients aged <30 years increased steadily from 2010 to 2018 (Figure 1B).

**Table 1:** Demographic and clinical features of 34297 HIV-infected patients in Hunan province.

**Characteristics**	**Category**	**No. of cases**
Gender	Male	25,338 (73.9%^*^)
	Female	8,918 (26.0%)
Age	<20 y	425 (1.2%^*^)
	20–30 y	5,070 (14.8%)
	30–40 y	6,707 (19.6%)
	40–50 y	7,778 (22.7%)
	≥50 y	14,150 (41.3%)
Calendar years	2003–2009	2,074 (6.1%)
	2010–2014	11,659 (34.1%)
	2015–2018	20,510 (59.8%)
Route of transmission	Homosexual route	5,519 (16.1%)
	Heterosexual route	24,397 (71.2%)
	Intravenous drug use	1,738 (5.1%)
	Other routes^&^	207 (0.6%)
	Unkclear routes	2,418 (7.1)
Region	Eastern Hunan	7,926 (23.1%)
	Western Hunan	5,681 (16.6%)
	Southern Hunan	10,564 (30.8%)
	Northern Hunan	4,257 (12.4%)
	Central Hunan	5,851 (17.1%)
Baseline CD4+ T-cell counts	<200 cells/μl	16,165 (47.2%)
	200–349 cells/μl	10,718 (31.3%)
	350–499 cells/μl	4,871 (14.2%)
	≥500 cells/μl	2,525 (7.4%)
Initial ART regimen	2NRTIs + NNRTIs	30,995 (90.4%)
	2NRTIs + INI	197 (0.6%)
	2NRTIs + PI	2,476 (7.2%)
	Others^#^	607 (1.8%)
Immunologic outcome	Poor immune reconstitution	3,962 (11.5%)
Clinical outcome	Under treatment	24„420 (71.2%)
	Lost to follow-up	3917 (11.4%)
	Therapy discontinued	1,531 (4.5%)
	Deaths	4,411 (12.9%)
	AIDS-related diseases	2,516 (7.3%)
	Accidental death	341 (1.0%)
	Suicide	143 (0.4%)
	Other	1,240 (3.6%)
	Undetermined	171 (0.5%)
Treatment switch	No	25,372 (74.0%)
	Yes	8,907 (26.0%)
	Side effects	2,618 (7.6%)
	First-line treatment failure	954 (2.8%)
	Drug interactions	227 (0.7%)
	Other reasons	1,588 (4.6%)
	Unclear reasons	3,520 (10.3%)

& *Other transmission routes included transfusion/blood products, and vertical transmission*.

* *The presence of missing data in the gender and age category*.

# *Other ART regimens included NRTI, 2NRTI, NRTI+PI, PI, NRTI+NNRTI, 3NRTI+PI, NRTI+INI+PI, NRTI+NNRTI+PI, 3NRTI, INI, NNRTI+PI*.

*ART, Antiretroviral treatment; NRTI, Nucleoside reverse transcriptase inhibitor; NNRTI, Non-Nucleoside reverse transcriptase inhibitor; INI, Integrase inhibitor; PI, Protease inhibitor*.

### Epidemiological Characteristics of New HIV Cases

#### Temporal Trend of New HIV Cases

**Figure 2:**
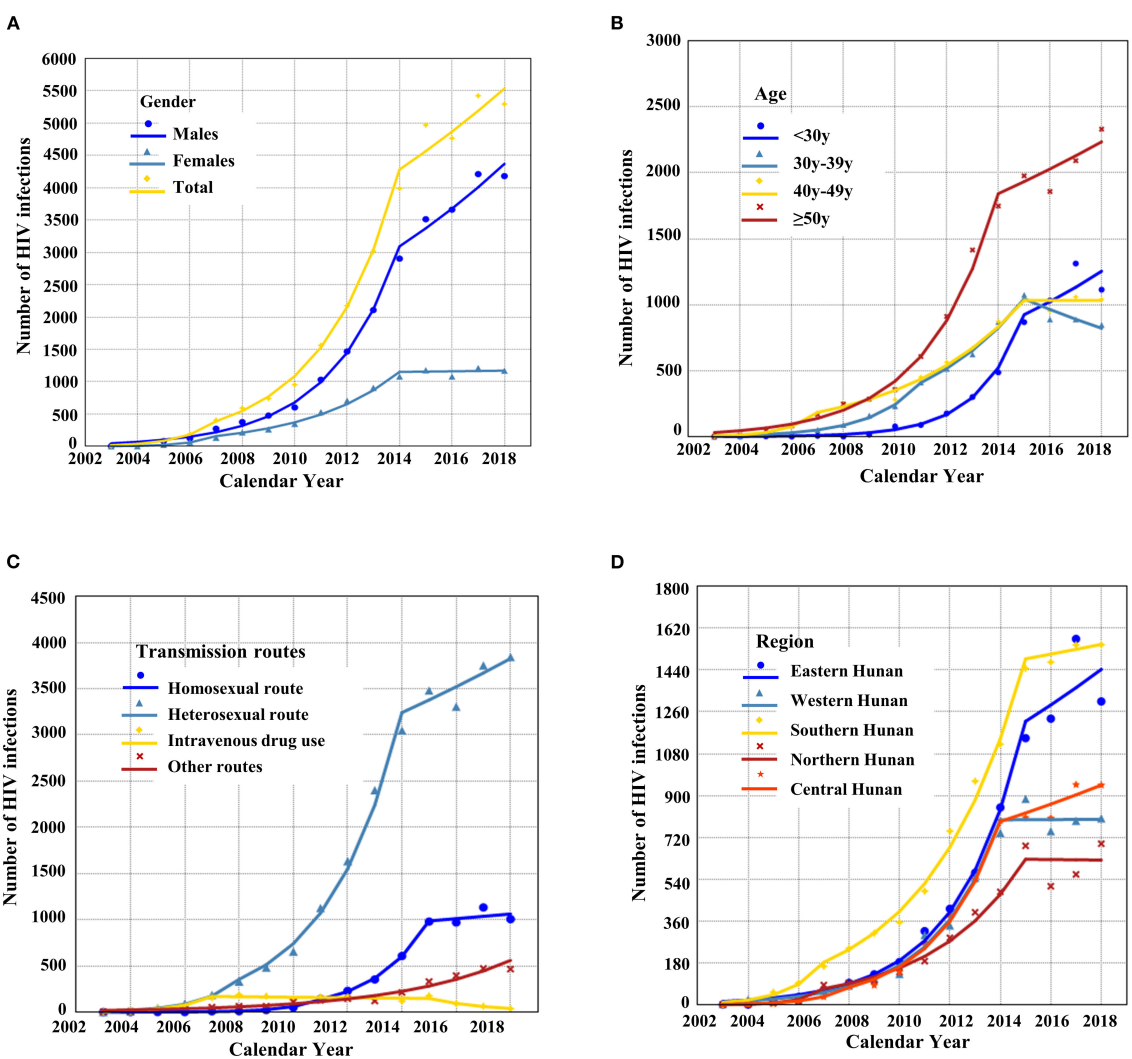
The temporal trend of the total number of HIV-1 infections **(A)** and HIV infections is stratified by gender **(A)**, age **(B)**, transmission **(C)**, and region **(D)**.

The proportion of new HIV cases was only 6.1% from 2003 to 2009, but a steady increase (34.1%, *N* = 11,659) was observed from 2010 to 2014. The majority of these new cases (*N* = 20,510, 59.8%) were observed from 2015 to 2018, indicating a substantial increase of new HIV cases during this period (Table 1). The Joinpoint regression estimated 46.1% of the average annual percentage change from 2003 to 2018 (AAPC_2003−2018_), suggesting the increasing number of HIV infections in Hunan province (Figure 2A). In contrast, the annual percentage change from 2004 to 2018 (APC_2014−2018_) was 6.62%, indicating a decreasing growth rate from 2014 to 2018 (Table 2). Although new HIV cases seemed to increase in both males and females, the number and the growth trend of HIV cases in males were significantly higher (Figure 2A). As shown in Figure 2B, the incidences of HIV new cases increased in all age groups (<30 years, 30–39 years, 40–49 years, ≥50 years). A significant increase in HIV infections was identified among patients <30 years (AAPC_2013−2018_: 60.9%) (Figure 2B).

**Table 2:** Temporal trend analysis of HIV cases stratified by gender, age, transmission routes, and geographical regions.

**Categories**	**Range of year**	**APC (95%CI)**	**Range of year**	**AAPC (95%CI)**
**Total**				
Total	2003–2007	113.1^*^ (34.4, 238.1)	2003–2018	46.1^*^ (31.3, 62.5)
	2007–2014	40.9^*^ (35.1, 47.0)		
	2014–2018	6.6^*^ (2.5, 10.9)		
**Gender**				
Male	2003–2014	46.1^*^ (40.2, 52.1)	2003–2018	35.1^*^ (31.2, 39.1)
	2014–2018	9.0^*^ (3.8, 14.5)		
Female	2003–2007	166.6^*^ (62.6, 337.3)	2003–2018	48.4^*^ (32.5, 66.2)
	2007–2014	32.7^*^ (27.7, 37.9)		
	2014–2018	0.4 (−3.8, 4.8)		
**Age**				
<30y	2003–2015	76.6^*^ (59.3, 95.9)	2003–2018	60.9^*^ (49.0, 73.7)
	2015–2018	10.7 (−1.5, 24.4)		
30–39y	2003–2011	68.3^*^ (54.6, 83.2)	2003–2018	38.3^*^ (32.3, 44.5)
	2011–2015	26.4^*^ (16.5, 37.2)		
	2015–2018	−7.7^*^ (−14.0, −0.9)		
40–49y	2003–2007	138.7^*^ (43.5, 296.9)	2003–2018	41.5^*^ (25.8, 59.1)
	2007–2015	24.1^*^ (19.6, 28.7)		
	2015–2018	0 (−8.7, 9.5)		
>50y	2003–2014	44.7^*^ (37.8, 51.8)	2003–2018	32.8^*^ (28.2, 37.5)
	2014–2018	4.9 (−1.4, 11.7)		
**Transmission routes**				
Homosexual	2003–2011	126.5^*^ (70.3, 201.2)	2003–2018	77.4^*^ (55.3, 102.6)
	2011–2015	64.2^*^ (44.4, 86.7)		
	2015–2018	2.5 (−5.2, 10.7)		
Heterosexual	2003–2008	104.1^*^ (44.6, 188.0)	2003–2018	48.7^*^ (34.6, 64.2)
	2008–2014	44.6^*^ (37.5, 52.1)		
	2014–2018	4.2^*^ (0.4, 8.2)		
Intravenous drug use	2003–2007	123.0^*^ (53.5, 224.0)	2003–2018	12.2^*^ (1.4, 24.1)
	2007–2015	−1.6 (−6.3, 3.2)		
	2015–2018	−36.3^*^ (−52.6, −14.3)		
Others	2003–2018	25.6^*^ (21.7, 29.6)	2003–2018	25.6^*^ (21.7, 29.6)
**Region**				
Eastern Hunan	2003–2015	45.1^*^ (37.6, 52.9)	2003–2018	36.2^*^ (30.5, 42.1)
	2015–2018	5.8 (−5.4, 18.2)		
Western Hunan	2003–2014	47.2^*^ (40.3, 54.4)	2003–2018	32.8^*^ (28.2, 37.5)
	2014–2018	0.1 (−6.2, 6.7)		
Southern Hunan	2003–2007	107.0^*^ (8.7, 294.3)	2003–2018	40.0^*^ (20.8, 62.3)
	2007–2015	30.0^*^ (24.8, 35.4)		
	2015–2018	1.4 (−7.2, 10.7)		
Northern Hunan	2003–2007	210.1^*^ (84.1, 422.3)	2003–2018	56.5^*^ (38.9, 76.4)
	2007–2015	31.7^*^ (27.5, 36.0)		
	2015–2018	−0.2 (−6.9, 7.1)		
Central Hunan	2003–2008	114.2^*^ (15.8, 296.3)	2003–2018	52.8^*^ (28.0, 82.3)
	2008–2014	48.3^*^ (37.1, 60.5)		
	2014–2018	4.6 (−0.9, 10.5)		

* *Indicates that the APC is significantly different from zero at the level of alpha = 0.05*.

#### The Spatial Disparity of HIV-Infected Patients

**Figure 3:**
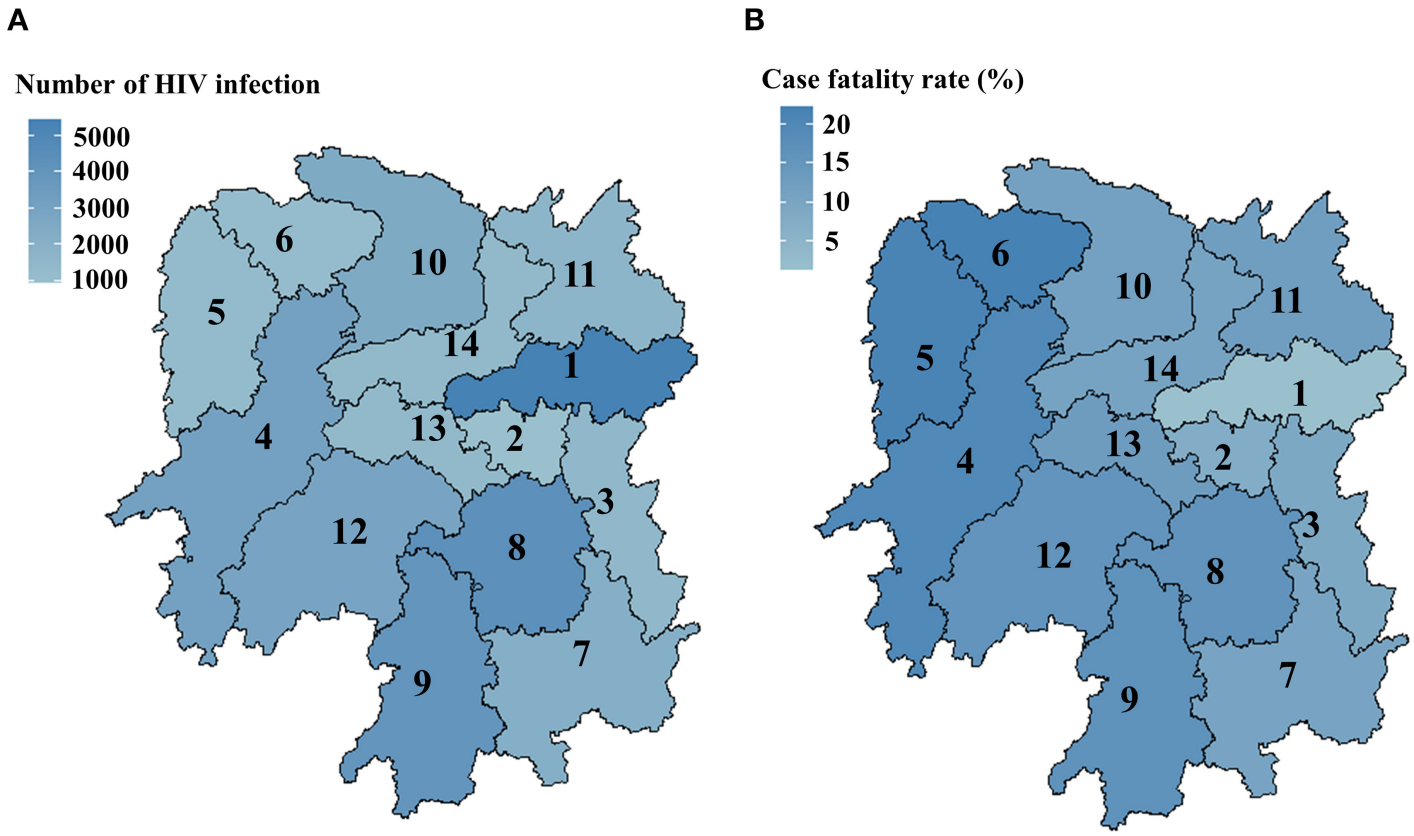
Geographical distribution maps of HIV-1 infections **(A)** and the case fatality rate **(B)** in Hunan province from 2003 to 2018. Eastern Hunan included region 1, region 2, and region 3; Western Hunan included region 4, region 5, and region 6; Southern Hunan included region 7, region 8, and region 9; Northern Hunan included region 10 and region 11; Central Hunan included region 12, region 13, and region 14.

We analyzed the spatial disparity of HIV-infected patients using geographical distribution maps (Figure 3). As shown in Table 1, the accumulated number of HIV infections (2003–2018) and HIV-associated fatality rates varied significantly between five geographical regions (Eastern Hunan, Southern Hunan, Western Hunan, Northern Hunan, Central Hunan). By 2018, Northern Hunan had the lowest number (*N* = 4,257) of HIV infections (Figure 3A). Western Hunan had the second-lowest number of HIV infections, but its case fatality rate was the highest (Figure 3B). Southern Hunan had the most cases of HIV infections and HIV-associated deaths. The growth rate of HIV infections in the above five regions varied (Figure 2D), but the growth trend slowed down or decreased from 2014 to 2018 (Supplementary Table S1).

#### Dynamic Changes in HIV Transmission Routes

From 2003 to 2005, intravenous drug use and blood transmission were the major transmission routes (>50%) of HIV infections in Hunan province. From 2006 to 2018, sexual transmission dominated the transmission route over time (Figure 1C). The absolute number of HIV infections through heterosexual transmission was far more common than that through other routes (Figure 2C). In 2018, the most common route of infection was heterosexual transmission (71.8%, *N* = 3,844), followed by homosexual transmission (18.8%, *N* = 1,007). Heterosexual transmission occurred mainly in male patients (67.2%, *N* = 16,414) and elderly patients aged ≥50 years (49.5%, *N* = 12,070). In contrast, the homosexual transmission was mostly found among young patients (age ≤30 years) (39.7%, *N* = 2,192) compared with elderly patients (14.6%, *N* = 806). Among all transmission routes, the homosexual transmission had the highest annual percentage change (Table 2).

### Clinical Characteristics and Outcomes of HIV Patients

#### ART Regimens

The ART regimen of two nucleoside reverse transcriptase inhibitors (NRTIs) plus one non-nucleoside reverse transcriptase inhibitor (NNRTI) was administered to a majority of HIV-infected patients (90.4%, *N* = 30,995). The most frequently prescribed NRTI backbones were a fixed combination of tenofovir and lamivudine (60.3%, *N* = 20,657). The most commonly prescribed third drug was an NNRTI such as efavirenz (68.2%, *N* = 23,380). Among 34,297 patients who received standard ART, 3,962 (11.5%) patients developed poor immune reconstitution. Moreover, 8,907 patients switched their treatment regimens because of adverse effects (29.3%, N-2618), followed by the first-line treatment failure (10.7%, *N* = 954), drug-drug interactions (227, 2.5%), and other reasons (Table 1).

#### CD4 Dynamics Before and After ART

**Figure 4:**
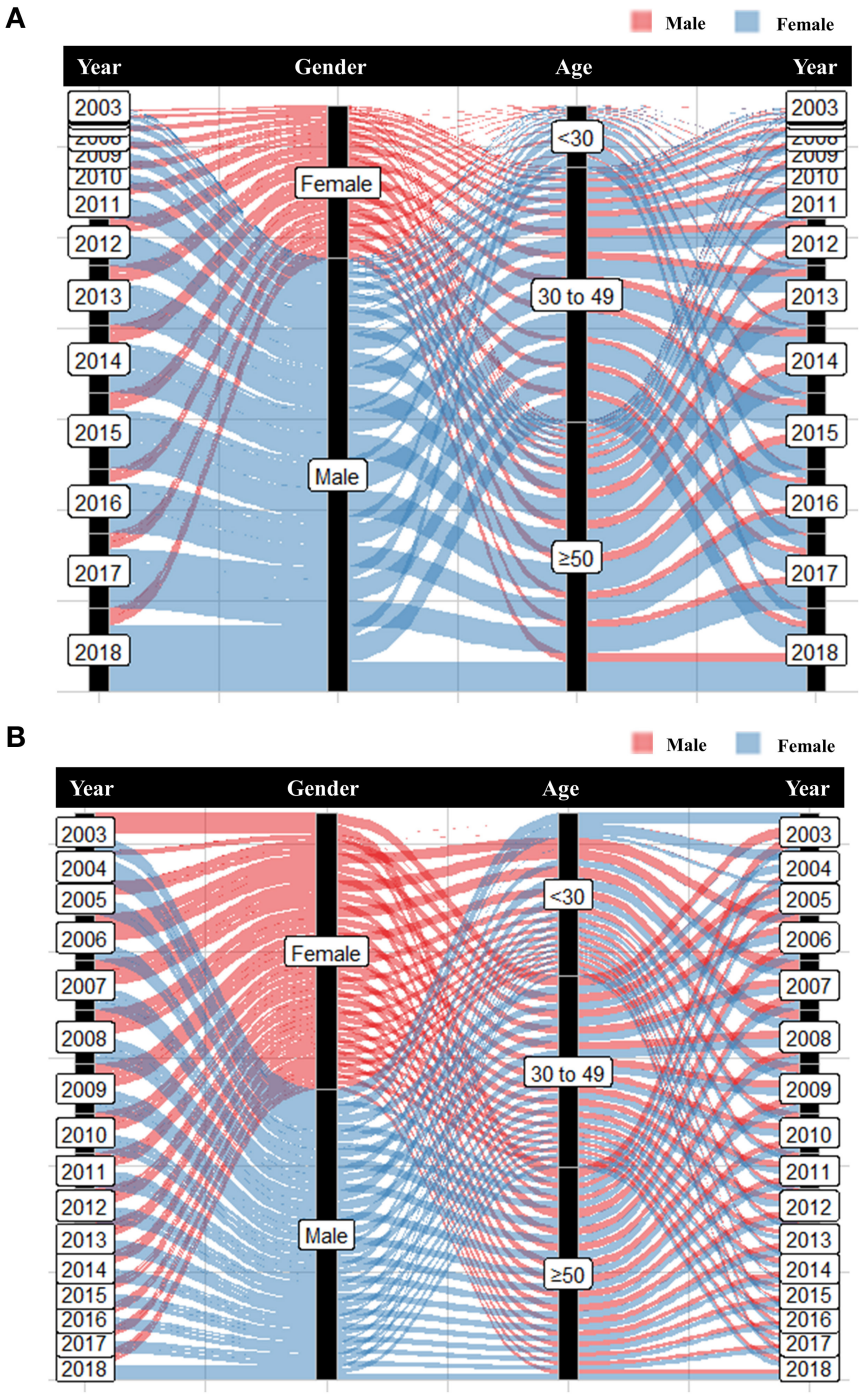
The distribution of advanced HIV disease in age and gender in Hunan Province from 2003 to 2018. **(A)** The number of advanced HIV disease from 2003 to 2018; **(B)** Proportions of advanced HIV disease from 2003 to 2018.

**Figure 5:**
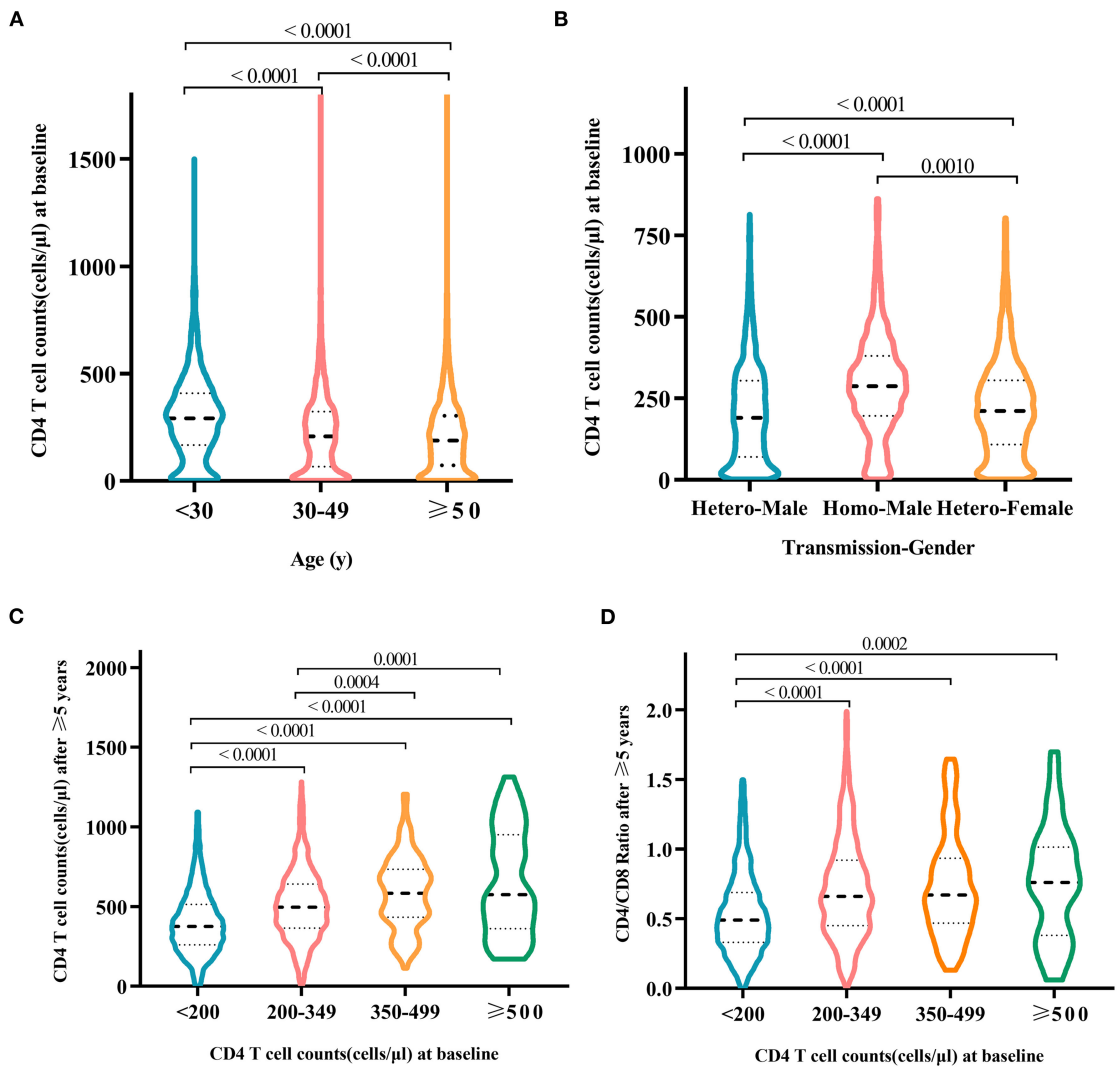
Differences in baseline CD4+ T-cell counts among HIV-infected patients at different ages **(A)** and patients of different genders infected by different transmission routes **(B)**. After treatment for more than 5 years, patients with different baseline CD4 counts were diagnosed with the follow-up of CD4 counts **(C)** and CD4/CD8 ratio **(D)**. Only statistically significant groups were labeled.

At baseline, a large proportion (47.2%, *N* = 16,165) of HIV-infected patients CD4+ T cell counts < 200 cells/μl at baseline (Table 1). From 2003 to 2018, there was a steady decline in the proportion of advanced HIV disease from 71.4% (2003) to 42.8% (2018) (Supplementary Figure S1). Although the number of advanced HIV disease increased over time, the proportion of advanced HIV disease from 2003 to 2018 showed decreasing patterns (Figure 4). In 2018, the proportion of advanced HIV disease was 31.9% in young patients aged <30 years (Supplementary Table S2). The median CD4+ T-cell counts at baseline were 212 cells/μl. CD4+ T-cell counts differed significantly in different age groups (Figure 5A) and elderly patients had lower CD4+ T-cell counts at baseline (elderly patients: 191.0 cells/μl, young patients: 294.6 cells/μl, *p*-value < 0.0001). As shown in Figure 5B, baseline CD4+ T-cell counts were higher in homosexual male patients (297.47 cells/μl), followed by heterosexual females (225.36 cells/μl), and heterosexual males (209.42 cells/μl).

After laboratory-confirmed diagnosis, HIV-positive patients received antiviral treatment with regular follow-up of CD4 counts every 6 months. First, CD4 counts increased significantly by comparing serum levels before treatment initiation and after 6-month treatment (252.9 vs. 349.1 cells/μl, *p*-value < 0.0001). Second, an average increase in CD4 levels of 105.5 cells/μl was observed among those patients who received treatment for 0.5–1 year. A significant increase in CD4 counts (452.2 cells/μl) was also observed among those patients treated for >5 years (Table 3). Third, higher levels of CD4 counts at baseline were associated with a higher increase in CD4 counts (Figure 5C) and CD4/CD8 ratio (Figure 5D; Supplementary Table S3). After >5 years of treatment, except for the patient group with baseline CD4 levels below 200 cells/μl, CD4 counts in other groups reached >500 cells/μl (Figure 5C). Those patients with baseline CD4 levels >200 cells/μl experienced the recovery of the CD4/CD8 ratio to 0.75 after >5 years of antiviral treatment (Figure 5D).

**Table 3:** Effects of antiretroviral therapy on CD4+ T-cell counts (cells/μl).

**Treatment time (year)**	**Patient number**	**CD4+** **T-cell counts**		**Increase (cells/μl)**	***t***	***P*-value**
		**Before ART**	**After ART**			
0.6–1	2,773	249.6	355.1	105.5	32.79	<0.001
1–2	3,896	269.1	397.2	128.1	42.63	<0.001
2–3	4,114	239.3	426.1	186.8	59.22	<0.001
3–4	3,115	226.0	440.9	215.0	58.03	<0.001
4–5	2,207	189.5	433.0	243.5	54.36	<0.001
>5	4,805	162.8	452.2	289.4	90.14	<0.001

#### Survival Time of HIV-Infected Patients Under ART

**Figure 6:**
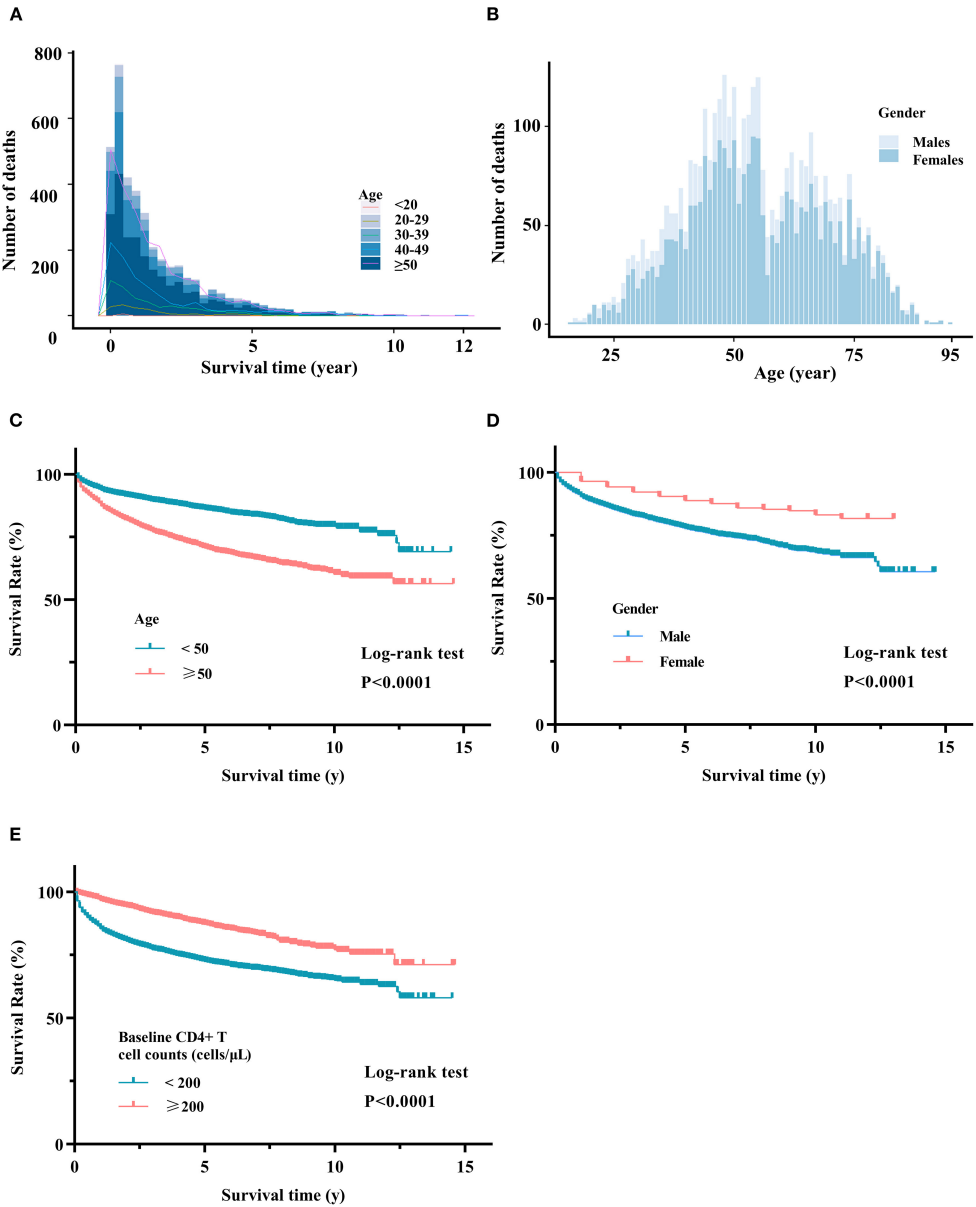
Survival analysis of HIV-associated death. **(A)** Distribution of HIV-associated death in different age groups. **(B)** Distribution of HIV-associated death in males and females. Kaplan-Meier curves in different age groups **(C)**, gender groups **(D)**, and baseline CD4 groups **(E)**. Log-rank tests were used to measure the statistical difference.

In our cohort of 34,297 HIV-infected patients, 24,420 (71.2%) patients were under treatment, 4,411 (12.9%) died, and 3,917 (11.4%) were lost to follow-up. Analysis of 4,411 deaths revealed AIDS-related illnesses (2,516, 57.0%), accidental deaths (341, 7.7%), suicides (143, 3.2%), or other causes (1,240, 28.1%) (Table 1). The highest proportion of deaths occurred in patients aged >50 years (Figure 6A).

**Figure 7:**
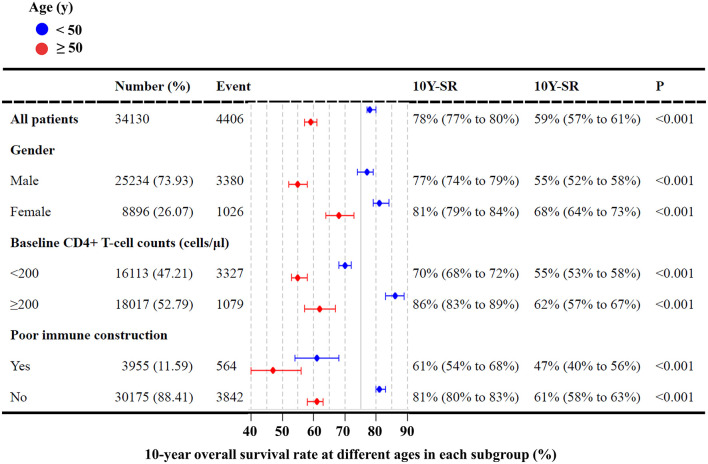
Survival rates of HIV-infected patients in different groups. 10Y-SR indicates the 10-year survival rate.

Our survival analyses revealed the survival rate of HIV-infected patients categorized by gender, age, region, transmission route, and baseline CD4 levels (Figures 6C–E). The overall 10-year survival rate was estimated to be 70.0%. The estimated 10-year survival rate was significantly higher in females vs. males (75.9 vs. 67.1%, *p*-value < 0.0001), patients <50 vs. ≥50 years (78.4 vs. 59.0%, *p*-value < 0.0001), and patients with baseline CD4 level >200 cells/μl vs. <200 cells/μl (76.9 vs. 63.2%, *p*-value < 0.0001) (Supplementary Table S6). As shown in Figure 7, our subgroup analyses revealed that the 10-year survival rate was significantly lower in older males (55%) than in young males (77%) and young females (81%). A low 10-year survival rate of 55% was also found in older patients with baseline CD4 levels <200 cells/μl.

## Discussion

Based on a large-scale cohort of 34,297 HIV-infected patients, our study presents the first survey of HIV infections (2003–2018) in Hunan province. Compared with other provinces in China, the HIV epidemic in Hunan province is not considered severe (30). Although there seems a growing number of HIV incidences, the overall growth rate in Hunan province has slowed down in recent years, implying the effectiveness of HIV prevention and control strategies. Most HIV infections in Hunan province are diagnosed in male patients, in agreement with other studies conducted from other provinces in China (27, 31). Our study finds that the majority of HIV infections in Hunan province are elderly patients aged ≥50 years, which is agreement with other national surveys (32–34). However, young males are considered the key population for HIV prevention given the increasing proportion of homosexual transmission. Although the mortality rate in young patients aged <30 years was relatively low, a significant increase in HIV infections can be traced to young populations, especially those high school and college students (21, 35).

Due to the long incubation period from HIV infection to the onset of AIDS and related symptoms, early HIV diagnosis remains difficult in the absence of routine screening (17). Our study showed that the proportion of HIV-infected patients with CD4+ T cell counts < 200 cells/μl at baseline was 42.8% in 2018 in Hunan Province, which was relatively higher than that in other regions (36, 37). In response to the national prevention strategies, free HIV testing services are currently available in Hunan to reduce the risk of advanced HIV disease. Early intervention, early detection, early control, and early treatment are strongly advocated in China, leading to better management of HIV infections in the future.

We observed a dynamic change in HIV transmission routes in the past decade. Before 2005, blood transmission and intravenous drug use are the main routes of transmission in China (16). To date, sexual transmission has become the predominant route of HIV transmission (22). Nevertheless, there is a growing trend of homosexual transmission across the country (25). In agreement with previous studies (22, 25), we found that homosexual transmission was more likely to occur in young males in Hunan province. We also observed a higher level of baseline CD4+ T-cell counts in male patients infected by homosexual transmission than those infected by heterosexual transmission, as well as a better immune outcome and a lower risk of deaths after ART. The possible reasons might be explained by the large proportion of young patients infected through homosexual transmission, and the proliferation of HIV drug resistance strains might be associated with transmission routes (38–44). For effective HIV prevention in China, more efforts are still needed to control both heterosexual and homosexual transmissions among high-risk populations.

Our study observed a difference in the case fatality rate among five geographical regions in Hunan province (Figure 3). The number of HIV infections was most in Southern Hunan, but the HIV-associated fatality rate was the highest in Western Hunan. This regional disparity might be explained by several reasons. (i) Economic and medical conditions are discrepant in different regions. (ii) Social stigma in different regions may play a role in HIV prevention and treatment. A recent study found that college students with higher education levels still hold negative attitudes toward HIV-infected patients (45). Previous studies suggest that HIV-related stigma and lack of social support have the potential to harm the health and wellbeing of HIV-infected patients (46, 47). (iii) HIV is known for its high genetic diversity (48), and the distribution of circulating HIV subtypes and strains exhibit regional differences (49–52). Further studies need to address the impact of diversified anti-HIV strategies and HIV strains on HIV prevention and treatment in different regions.

It is known that wide applications of HIV prevention and treatment strategies have reduced HIV-associated mortality and morbidity, not only in China but also in many other countries (53). We estimated 70.0% of the overall 10-year survival rate of treated patients using a large-scale cohort in Hunan province. Compared with other patient groups, elderly males in Western Hunan had a lower survival rate (Figure 6). It is known that HIV infection can impair the human immune system (54), and the elderly are more vulnerable to infectious diseases because aging can affect the innate and adaptive immune system (55). As expected, we observed an age-related decrease in CD4 levels at baseline. In agreement with previous studies (56, 57), we found that baseline CD4 levels were associated with the recovery of CD4 levels and CD4/CD8 after ART. Furthermore, elderly patients are unlikely to achieve the same virological and immune responses as young patients, probably due to the low CD4 levels at baseline and relatively poor immune function (58).

There are limitations to our study. First, our database included the majority (>70%) of laboratory-confirmed HIV-infected patients in Hunan province, but this official database does not include undiagnosed cases or those immigrant patients who were not designated to local health facilities in Hunan province. Second, our database only included HIV-associated factors, but not other factors such as ethnicity, occupation, degree of education, marital status, or traveling history. Therefore, our analysis cannot address all potential risk factors associated with HIV prevention and treatment. Third, our retrospective study could not reveal clinical efficacies of ART because HIV-infected patients were not randomized and treatment switch is often considered at different timepoints in clinical practice. Fourth, our study only focused on HIV epidemiology in Hunan Province, and future studies need to report HIV epidemiology from a global perspective.

## Conclusion

This study reveals epidemiological and clinical characteristics of HIV-infected patients in Hunan province, shedding light on the focus of HIV prevention and treatment in certain high-risk populations and geographical regions. Although the overall growth rate of new HIV cases slows down in Hunan province, a special focus should be taken on elderly males who were infected mainly by heterosexually transmission. Furthermore, a high proportion of advanced HIV disease indicates the importance of HIV routine testing and surveillance. Better management strategies are still needed to effectively control the spread of HIV infections from a regional and global perspective.

## Data Availability Statement

The raw data supporting the conclusions of this article will be made available after the approval of the Ethics Committees.

## Ethics Statement

The studies involving human participants were reviewed and approved by the Ethics Committees of The First Hospital of Changsha (Approval ID: 202160). Written informed consent from the participants' legal guardian/next of kin was not required to participate in this study in accordance with the national legislation and the institutional requirements.

## Author Contributions

TY performed statistical analyses and drafted the manuscript. PZ, TYH, and ED contributed with data interpretation and discussions of the manuscript. JH, JZ, and FZ performed data acquisition. XYH and FZ supervised the study. GL obtained funding and revised the manuscript. All authors contributed to the final article. All authors contributed to the article and approved the submitted version.

## Funding

This research was funded by the National Nature Science Foundation of China (31871324, 81730064, and 31571368), and the National Science and Technology Major Project (2018ZX10715004). The funders had no role in the study design, data collection, data analysis, data interpretation, or writing of the report.

## Conflict of Interest

The authors declare that the research was conducted in the absence of any commercial or financial relationships that could be construed as a potential conflict of interest.

## Publisher's Note

All claims expressed in this article are solely those of the authors and do not necessarily represent those of their affiliated organizations, or those of the publisher, the editors and the reviewers. Any product that may be evaluated in this article, or claim that may be made by its manufacturer, is not guaranteed or endorsed by the publisher.

## Abbreviations

AIDS: Acquired immune deficiency syndrome
AAPC: Annual percentage changes
APC: Annual percentage changes
ART: Antiretroviral therapies
HIV: Human immunodeficiency virus
INI: Integrase inhibitor
MSM: Men who sex with men
NFATP: The National Free Antiretroviral Treatment Program
NNRTIs: Non-Nucleoside reverse transcriptase inhibitors
NRTI: Nucleoside reverse transcriptase inhibitor.
