# Supplementary material for: Epidemiology and Clinical Outcomes of HIV Infection in South-Central China: A Retrospective Study From 2003 to 2018
Source: Front Public Health. 2022 Jun 9;10:902537. doi: 10.3389/fpubh.2022.902537 (PMC9218543; doi:10.3389/fpubh.2022.902537)
Supplement: Supplementary file 1 [file Table_1.DOCX]

Supplementary Material

# Supplementary Tables

**Table S1. Temporal trend of the number of new HIV-infected cases in 14 regions of Hunan province from 2003 to 2018.**

| **Categories** | **Range of Year** | **APC^&^ (95%CI)** | **Range of Year** | **APC (95%CI)** |
| --- | --- | --- | --- | --- |
| **Region1** | 2003 to 2016 | 39.9* (35.7, 44.3) | 2003 to 2018 | 33.0* (29.0, 37.1) |
|  | 2016 to 2018 | -4.4 (-18.6, 12.1) |  |  |
| **Region2** | 2003 to 2015 | 47.9* (34.9, 62.2) | 2003 to 2018 | 36.9* (27.1, 47.5) |
|  | 2015 to 2018 | 0.6 (-17.5, 22.6) |  |  |
| **Region3** | 2003 to 2013 | 65.8* (42.7, 92.5) | 2003 to 2018 | 46.5* (33.6, 60.6) |
|  | 2013 to 2018 | 14.5* (5.4, 24.3) |  |  |
| **Region4** | 2003 to 2014 | 47.1* (39.3, 55.5) | 2003 to 2018 | 33.9* (28.6, 39.3) |
|  | 2014 to 2018 | 3.2 (-4.2, 11.1) |  |  |
| **Region5** | 2003 to 2011 | 85.0* (55.7, 119.7) | 2003 to 2018 | 50.3* (37.0, 64.7) |
|  | 2011 to 2014 | 45.7* (10.3, 92.6) |  |  |
|  | 2014 to 2018 | 1.6 (-4.6, 8.2) |  |  |
| **Region6** | 2003 to 2014 | 36.2* (22.3, 51.7) | 2003 to 2018 | 22.2* (12.4, 32.9) |
|  | 2014 to 2018 | -9.3 (-25.0, 9.6) |  |  |
| **Region7** | 2003 to 2014 | 46.1* (34.9, 58.3) | 2003 to 2018 | 36.1* (28.6, 44.0) |
|  | 2014 to 2018 | 11.9* (1.8, 22.9) |  |  |
| **Region8** | 2003 to 2007 | 100.2* (2.3, 291.8) | 2003 to 2018 | 30.6* (11.8, 52.5) |
|  | 2007 to 2015 | 17.8* (12.3, 23.5) |  |  |
|  | 2015 to 2018 | -2.7 (-14.8, 11.0) |  |  |
| **Region9** | 2003 to 2013 | 76.0* (60.8, 92.5) | 2003 to 2018 | 50.1* (42.1, 58.6) |
|  | 2013 to 2018 | 9.2* (4.6, 14.0) |  |  |
| **Region10** | 2003 to 2014 | 45.3* (33.3, 58.3) | 2003 to 2018 | 33.3* (25.4, 41.6) |
|  | 2014 to 2018 | 5.1 (-4.9, 16.3) |  |  |
| **Region11** | 2003 to 2007 | 174.1* (46.4, 413.2) | 2003 to 2018 | 47.4* (27.5, 70.4) |
|  | 2007 to 2015 | 26.1* (20.2, 32.4) |  |  |
|  | 2015 to 2018 | -2.4 (-13.0, 9.6) |  |  |
| **Region12** | 2003 to 2014 | 53.9* (42.5, 66.2) | 2003 to 2018 | 38.3* (31.1 45.9) |
|  | 2014 to 2018 | 3.1 (-4.6, 11.5) |  |  |
| **Region13** | 2003 to 2014 | 56.5* (44.3, 69.8) | 2003 to 2018 | 39.4* (31.7, 47.5) |
|  | 2014 to 2018 | 1.4 (-6.9, 10.3) |  |  |
| **Region14** | 2003 to 2007 | 114.3* (40.6, 226.6) | 2003 to 2018 | 49.0* (35.2, 64.3) |
|  | 2007 to 2013 | 48.6* (41.0, 56.7) |  |  |
|  | 2013 to 2018 | 11.8* (9.2, 14.6) |  |  |
| **Total** | 2003 to 2007 | 113.1* (34.4, 238.1) | 2003 to 2018 | 46.1* (31.3, 62.5) |
|  | 2007 to 2014 | 40.9* (35.1 47.0) |  |  |
|  | 2014 to 2018 | 6.6* (2.5, 10.9) |  |  |

&: APC, Annual percentage change.

* Indicates that the APC is significantly different from zero at the alpha=0.05 level.

**Table S2. Proportions of advance HIV diseases (CD4+ T cell counts < 200 cells/μl) of HIV infections in Hunan Province, by age and gender, from 2003 to 2018.**

| **Year** | **Total** | **Delayed Diagnosis** | **Proportion（%）** | **Total** | **Delayed Diagnosis** | **Proportion（%）** | **Total** | **Delayed Diagnosis** | **Proportion（%）** |
| --- | --- | --- | --- | --- | --- | --- | --- | --- | --- |
|  | **<30 years** | | | **30 to 49 years** | | | **≥50 years** | | |
| **Males** | | | | | | | | | |
| 2003 | 1 | 1 | 100 | 1 | 0 | 0 | 2 | 1 | 50.00 |
| 2004 | 2 | 2 | 100 | 16 | 12 | 75.00 | 10 | 7 | 70.00 |
| 2005 | 3 | 1 | 33.33 | 38 | 26 | 68.42 | 41 | 28 | 68.29 |
| 2006 | 0 | 0 | 0 | 64 | 52 | 81.25 | 61 | 54 | 88.52 |
| 2007 | 6 | 4 | 66.67 | 153 | 114 | 74.51 | 114 | 90 | 78.95 |
| 2008 | 1 | 1 | 100 | 209 | 171 | 81.82 | 166 | 139 | 83.73 |
| 2009 | 10 | 8 | 80.00 | 272 | 195 | 71.69 | 196 | 145 | 73.98 |
| 2010 | 51 | 24 | 47.06 | 305 | 204 | 66.89 | 243 | 174 | 71.60 |
| 2011 | 55 | 28 | 50.91 | 567 | 331 | 58.38 | 409 | 267 | 65.28 |
| 2012 | 109 | 42 | 38.53 | 731 | 427 | 58.41 | 626 | 414 | 66.13 |
| 2013 | 214 | 72 | 33.64 | 914 | 470 | 51.42 | 980 | 611 | 62.35 |
| 2014 | 390 | 109 | 27.95 | 1277 | 583 | 45.65 | 1234 | 677 | 54.86 |
| 2015 | 743 | 173 | 23.28 | 1631 | 673 | 41.26 | 1410 | 768 | 54.47 |
| 2016 | 878 | 227 | 25.85 | 1444 | 562 | 38.92 | 1336 | 601 | 44.99 |
| 2017 | 1087 | 354 | 32.57 | 1511 | 580 | 38.39 | 1554 | 696 | 44.79 |
| 2018 | 972 | 305 | 31.38 | 1495 | 690 | 46.15 | 1702 | 800 | 47.00 |
| **Females** | | | | | | | | | |
| 2003 | 0 | 0 | 0 | 2 | 2 | 100 | 1 | 1 | 100 |
| 2004 | 0 | 0 | 0 | 0 | 0 | 0 | 2 | 1 | 50.00 |
| 2005 | 0 | 0 | 0 | 20 | 16 | 80 | 4 | 2 | 50.00 |
| 2006 | 1 | 1 | 100 | 42 | 30 | 71.43 | 22 | 19 | 86.36 |
| 2007 | 1 | 1 | 100 | 78 | 55 | 70.51 | 55 | 39 | 70.91 |
| 2008 | 1 | 1 | 100 | 133 | 95 | 71.43 | 82 | 60 | 73.17 |
| 2009 | 6 | 5 | 83.33 | 167 | 111 | 66.47 | 90 | 71 | 78.89 |
| 2010 | 27 | 17 | 62.96 | 207 | 122 | 58.94 | 114 | 76 | 66.67 |
| 2011 | 34 | 15 | 44.12 | 296 | 161 | 54.39 | 199 | 115 | 57.79 |
| 2012 | 68 | 26 | 38.24 | 349 | 214 | 61.32 | 286 | 177 | 61.89 |
| 2013 | 87 | 36 | 41.38 | 381 | 202 | 53.02 | 436 | 236 | 54.13 |
| 2014 | 98 | 31 | 31.63 | 468 | 217 | 46.37 | 515 | 250 | 48.54 |
| 2015 | 126 | 31 | 24.60 | 483 | 188 | 38.92 | 568 | 256 | 45.07 |
| 2016 | 155 | 47 | 30.32 | 399 | 142 | 35.59 | 523 | 202 | 38.62 |
| 2017 | 226 | 78 | 34.51 | 438 | 171 | 39.04 | 539 | 193 | 35.81 |
| 2018 | 143 | 51 | 35.66 | 394 | 183 | 46.45 | 630 | 253 | 40.16 |

**Table S3. Analysis of CD4+ T-cell counts and CD4/CD8 ratio during the anti-HIV treatment.**

| **Duration of treatment (years)** | **Clinical parameters** | **Baseline CD4+ T cell counts (cells/μl)** | | | | **F** | **P** |
| --- | --- | --- | --- | --- | --- | --- | --- |
|  |  | **<200** | **200-350** | **350-499** | **≥500** |  |  |
| **0.5 ≤ t < 2** | CD4 level (cells/μl) | 232.24 | 405.77 | 533.04 | 656.07 | 1143.39 | <0.001 |
|  | CD4/CD8 ratio | 0.36 | 0.56 | 0.68 | 0.76 | 174.63 | <0.001 |
| **2 ≤ t < 5** | CD4 level (cells/μl) | 321.65 | 480.75 | 580.81 | 720.16 | 828.42 | <0.001 |
|  | CD4/CD8 ratio | 0.49 | 0.68 | 0.76 | 0.83 | 162.24 | <0.001 |
| **t ≥5** | CD4 level (cells/μl) | 406.09 | 515.66 | 587.01 | 646.18 | 111.42 | <0.001 |
|  | CD4/CD8 ratio | 0.60 | 0.74 | 0.75 | 0.76 | 25.24 | <0.001 |

**Table S4. Analysis of CD4+ T-cell counts and CD4/CD8 ratio in the context of different transmission routs and gender.**

| **Duration of treatment** | **Clinical parameters** | **Category^*^** | | | **F** | **P** |
| --- | --- | --- | --- | --- | --- | --- |
|  |  | **Ho-Male** | **He-Male** | **He-Female** |  |  |
| **0.5 ≤ t < 2 years** | Baseline CD4 counts (cells/μl) ^&^ | 324.43 | 233.61 | 272.48 | 65.24 | <0.001 |
|  | CD4 (cells/μl) | 483.25 | 345.90 | 396.29 | 86.99 | <0.001 |
|  | CD4/CD8 ratio | 0.58 | 0.49 | 0.62 | 10.84 | <0.001 |
| **2 ≤ t < 5 years** | Baseline CD4 counts (cells/μl) | 280.26 | 204.00 | 226.53 | 156.65 | <0.001 |
|  | CD4 counts (cells/μl) | 508.68 | 400.41 | 508.68 | 144.2 | <0.001 |
|  | CD4/CD8 ratio | 0.62 | 0.58 | 0.69 | 23.53 | <0.001 |
| **t ≥ 5 years** | Baseline CD4 level (cells/μl) | 226.09 | 149.15 | 169.52 | 87.07 | <0.001 |
|  | CD4 (cells/μl) | 484.67 | 425.55 | 509.88 | 42.38 | <0.001 |
|  | CD4/CD8 ratio | 0.74 | 0.66 | 0.75 | 4.84 | 0.008 |

&: Baseline CD4+ T-cell levels in each age group with different treatment durations.

*Ho-Male: male infections who was infected by homosexual transmission.

*He-Male: male infections who was infected by heterosexual transmission.

*He-Female: female infections who was infected by heterosexual transmission.

**Table S5. Analysis of CD4+ T-cell counts and CD4/CD8 ratio in the context of age.**

| **Duration of treatment** | **Clinical parameters** | **Age** | | | **F** | **P** |
| --- | --- | --- | --- | --- | --- | --- |
|  |  | **<30y** | **30-49y** | **≥50y** |  |  |
| **0.5 ≤t< 2 years** | Baseline CD4 level (cells/μl) ^*^ | 317.97 | 232.75 | 212.00 | 53.90 | <0.001 |
|  | CD4 (cells/μl) | 483.86 | 369.03 | 317.31 | 83.87 | <0.001 |
|  | CD4/CD8 ratio | 0.57 | 0.48 | 0.51 | 7.94 | <0.001 |
| **2≤t< 5 years** | Baseline CD4 level (cells/μl) | 286.31 | 222.05 | 201.88 | 143.53 | <0.001 |
|  | CD4 (cells/μl) | 510.76 | 456.61 | 382.10 | 180.48 | <0.001 |
|  | CD4/CD8 ratio | 0.61 | 0.60 | 0.63 | 2.27 | 0.103 |
| **t ≥ 5 years** | Baseline CD4+ T cell count (cells/μl) | 227.46 | 164.63 | 149.07 | 36.27 | <0.001 |
|  | CD4 (cells/μl) | 489.84 | 471.42 | 416.91 | 32.74 | <0.001 |
|  | CD4/CD8 ratio | 0.68 | 0.68 | 0.69 | 0.03 | 0.970 |

*: Baseline CD4+ T-cell levels in each age group with different treatment durations.

**Table S6. Estimated survival rate after antiretroviral treatment.**

| **Category** | **1 Year** | **3 Years** | **5 Years** | **7 Years** | **10 Years** |
| --- | --- | --- | --- | --- | --- |
| **All** | | | | | |
| **All** | 0.897 (0.893,0.901) | 0.828 (0.823,0.834) | 0.783 (0.776,0.790) | 0.749 (0.741,0.758) | 0.700 (0.686,0.714) |
| **Gender** | | | | | |
| **Male** | 0.889 (0.884,0.894) | 0.815 (0.809,0.821) | 0.765 (0.757,0.773) | 0.728 (0.718,0.739) | 0.671 (0.652,0.690) |
| **Female** | 0.918 (0.911,0.924) | 0.862 (0.853,0.871) | 0.825 (0.815,0.836) | 0.796 (0.783,0.810) | 0.759 (0.738,0.781) |
| **Age** | | | | | |
| **<50** | 0.926 (0.922,0.931) | 0.882 (0.877,0.888) | 0.850 (0.843,0.858) | 0.824 (0.814,0.833) | 0.784 (0.768,0.800) |
| **≥50** | 0.856 (0.849,0.862) | 0.754 (0.745,0.763) | 0.692 (0.680,0.703) | 0.648 (0.634,0.663) | 0.590 (0.566,0.614) |
| **Baseline CD4+ T cell (cells/μl)** | | | | | |
| **<200** | 0.831 (0.824,0.837) | 0.750 (0.742,0.758) | 0.704 (0.694,0.713) | 0.674 (0.664,0.685) | 0.632 (0.616,0.648) |
| **≥200** | 0.964 (0.960,0.967) | 0.910 (0.904,0.916) | 0.869 (0.860,0.878) | 0.829 (0.816,0.843) | 0.769 (0.741,0.798) |

# Supplementary Figure


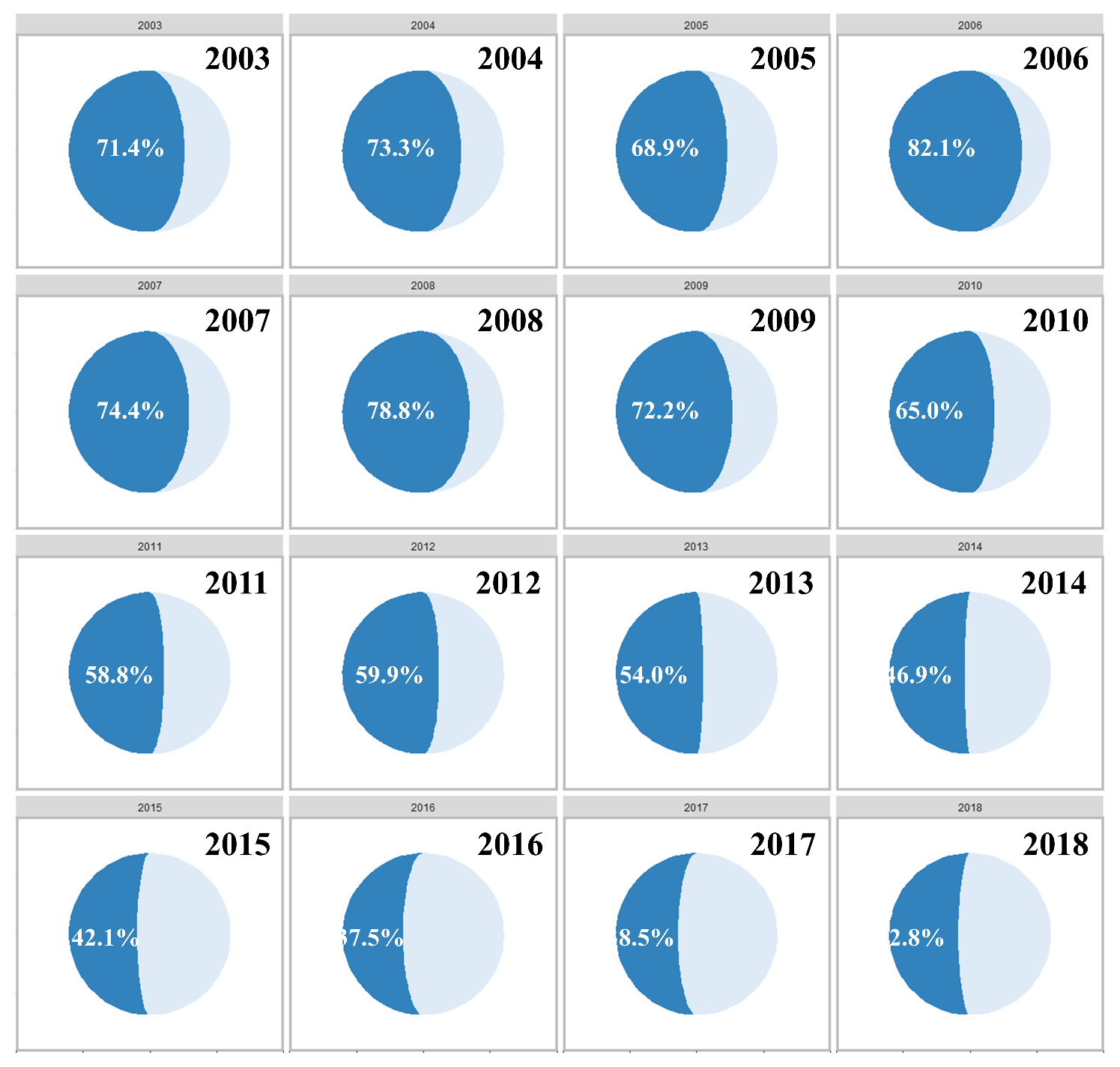
 **Figure S1.** Proportions of advanced HIV disease (CD4+ T cell counts < 200 cells/μl) from 2003 to 2018. The area in dark blue represents the proportion of delayed diagnosis.
